# Psoas Muscle Index Defined by Computer Tomography Predicts the Presence of Postoperative Complications in Colorectal Cancer Surgery

**DOI:** 10.3390/medicina57050472

**Published:** 2021-05-11

**Authors:** Zalán Benedek, Szabolcs Todor-Boér, Loránd Kocsis, Orsolya Bauer, Nicolae Suciu, Marius Florin Coroș

**Affiliations:** 1 Surgical Clinic Mureș County Clinical Hospital, “G.E. Palade” University of Medicine, Pharmacy, Science and Technology of Târgu Mureş, 540142 Târgu Mureș, Romania; benedek.zalan@gmail.com (Z.B.); orsolyabauer@gmail.com (O.B.); suciu_nicolae_mg@yahoo.com (N.S.); mcoros@gmail.com (M.F.C.); 2 Surgical Clinic Mureș County Clinical Hospital, 540103 Târgu Mureș, Romania; 3 Department of Anatomy and Embryology, “G.E. Palade” University of Medicine, Pharmacy, Science and Technology of Târgu Mureş, 540142 Târgu Mureș, Romania; lorand.kocsis@umfst.ro

**Keywords:** colorectal cancer, psoas muscle index, Clavien-Dindo classification, sarcopenia

## Abstract

*Background and Objectives*: Sarcopenia is a recognized prognostic factor for both complications and survival in cancer patients. This study aims to analyze the relationship between sarcopenia measured by psoas muscle index on computer tomography scans and the presence of postoperative complications in colorectal cancer surgery. *Materials and Methods*: In a prospective study we recorded data from 51 patients who underwent colorectal cancer surgery in the Mures County Clinical Hospital, Romania. Total psoas muscle area and psoas density were measured at the level of the third lumbal vertebra (L3) for further index calculation. We also evaluated the general characteristics and laboratory analyses to obtain more information about status of the patients. Short-term postoperative complications were scored according to the Clavien-Dindo classification. *Results*: The majority of the 51 patients were male (61%) and the median age was 65 years. More than half of the cancer was located in the rectum (56.9%), a quarter in the right colon (25.5%), the rest in the sigmoid (11.8%), and the left colon (5.9%). Twenty-one patients (41.2%) developed a complication, five (9.8%) of these were Clavien-Dindo grade 3, 4 or 5 (high grade) and sixteen (31.3%) grade 1 or 2 (low grade). The low- and high-grade groups showed a significantly lower right psoas muscle area, left psoas muscle area, total psoas muscle area, and psoas muscle index (*p* < 0.001 in all cases). Among laboratory analyses, a significantly lower perioperative hematocrit, hemoglobin, and albumin level were found in patients who developed complications. Furthermore we observed that an elevated serum C-reactive protein level was associated with a higher grade of complication (*p* < 0.043). *Conclusions*: The psoas muscle index (PMI) influence on the postoperative outcome is an important factor in our single center prospective study and it appears to be a good overall predictor in colorectal surgery. A lower PMI is directly associated with a low or high grade complication by Clavien-Dindo classification. Perioperative inflammatory and nutritional status evidenced by serum C-reactive protein (CRP) and albumin level influences the presence of postoperative complications.

## 1. Introduction

Colorectal cancer (CRC) is one of the most common cause of cancer related death. According to GLOBOCAN data it is the third most deadly and the fourth most commonly diagnosed malignant disease and it’s incidence has an increasing tendency [1,2]. The prognosis in CRC is highly influenced by clinical parameters as bowel obstruction and pathologic factors (tumor stage, presence of neurovascular invasion, lymph node status, etc.) [3]. The functional status and body composition are also important factors and the modification of these factors can influence the outcome of oncological patients. The loss of skeletal muscle mass and function or sarcopenia are characteristic for elderly patients, but some diseases can also develop and can warn of an ongoing clinical problem [4]. Psoas cross-sectional area provides an estimation of overall muscle mass and has been used in several studies to predict lean muscle mass [5]. A few studies defined sarcopenia based on measuring psoas muscle area at the level of the third lumbar vertebra (L3) and calculating psoas muscle index (PMI) without using any additional software. It has been proved that the incidence of sarcopenia in cancer patients is higher than in healthy patients with rates between 15 and 40% [6,7]. Sarcopenia identified by computer tomography (CT) scan as a gold standard for it, which is associated with impaired overall survival in gastrointestinal malignancies and postoperative morbidity, is more frequent in colorectal cancer patients in both non-metastatic and metastatic diseases [8]. CT scan defined PMI is an imaging marker of sarcopenia. In presence of sarcopenia, the ideal should be to screen all the patients before colorectal surgery and discuss with them the possible negative effect and impact of sarcopenia during postoperative care [9,10].

This study aims to demonstrate the impact of the preoperative CT scan-based PMI on postoperative complications defined by Clavien-Dindo classification following elective surgery for colorectal cancer.

## 2. Materials and Methods

### 2.1. Study Design and Data Collection

We conducted a prospective study in Mures County Clinical Hospital between 2019 October and 2020 March. This study included patients who underwent potentially curative elective colorectal surgery for primary colorectal cancer. We excluded from our study patients with existing inflammatory conditions or recurrent oncological disease. All patients received preoperative medication, antibiotic- and thromboprophylaxis before anesthesia induction. On admission, all the laboratory analyses and CT scan results were collected prospectively in a database. A surgical team clinically assessed all the patients on each postoperative day to identify any postoperative complication. Data regarding the complications were classified retrospectively by the Clavien-Dindo classification.

### 2.2. Patient and Perioperative Factors

Patient data such as gender, age, body mass index (BMI), main symptoms; laboratory parameters as complete blood count, albumin and protein level, biochemical profile, C-reactive protein (CRP), and erythrocyte sedimentation rate (ESR) were assessed.

### 2.3. Radiological Measurements

**Figure 1:**
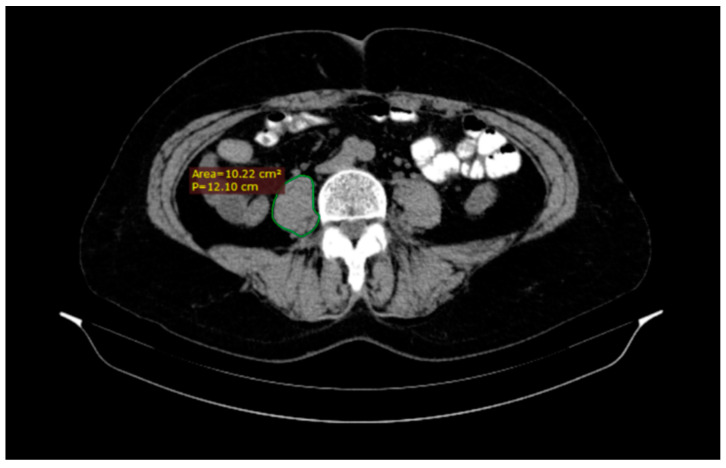
A quick tracing of the right (R) psoas muscle at the third lumbar vertebra (L3) level using DICOM Viewer software which automatically generates the area.

A radiologist analyzed all CT scans on a standard desktop computer screen using Radiant DICOM Viewer (Medixant, Poznan, Poland) and IQ View (Precision Diagnostic Systems, Inc., Landrose, USA) software. We used the CT images to assess the psoas muscle area and psoas density for each patient (Figure 1). The level of the L3 vertebra was selected as a landmark on the cross-sectional horizontal image and the psoas muscle area was traced (cm^2^) as the region of interest of the iliopsoas muscle contour. The total psoas area was calculated by the sum of the left and right psoas area, and then normalized by using each patient’s height to produce a PMI in cm^2^/m^2^. At the same time, the mean psoas muscle density (right psoas muscle density + left psoas muscle density/2) was measured in Hounsfield Unit (HU) [11,12].

### 2.4. Postoperative Follow-Up and Complications by Clavien-Dindo Classification

In the postoperative period, all the patients were assessed two times per day to identify postoperative complications. The postoperative complications were classified according to the validated Clavien-Dindo classification, taking into account the nature, severity and management of complications (Table 1) We also grouped them into three categories: no complications (patients without postoperative complications), low grade (grade 1 or 2; minor postoperative complications) and high grade (grade 3, 4, and 5; major postoperative complications) [13,14].

### 2.5. Statistical Analyses

Descriptive and inferential statistics were performed. The normality of distribution of continuous variables was tested by the Shapiro-Wilk test; no variable followed a normal distribution. Continuous variables were expressed as median (25th percentile, 75th percentile) and the medians were compared using the Kruskal-Wallis test. Categorical variables were displayed as frequencies, n (%), and between-group comparisons were performed using the Chi-square test. Logistic regression analysis was also performed to determine the odds ratio (OR) of the grade of complication. A value of *p* < 0.05 was considered significant. The IBM SPSS Statistics 22 program (IBM Corp., Armonk, NY, USA) software was used for the statistical analyses of the data.

## 3. Results

In our study 51 patients were included who were diagnosed with colorectal cancer and each of them underwent elective colorectal surgery with radical intent. 62% of the patients were male and the median age was 65 years (Interquartile range (IQR): 56–71). In more than half of the cases the tumor was located at the level of the rectum (56.9%), 25.5% at level of the right colon, the rest in the sigmoid colon (11.8%), and the left colon (5.9%). The results are summarized in Table 2.

Twenty-one patients (41.2%) developed postoperative complication, most of them low grade (16 patients), and were treated conservatively with antiemetics, analgetics. High grade complications were observed in 5 patients requiring significant intervention. The Clavien-Dindo complication grade is summarized in Table 3.

The relationship between postoperative complication grade and the recorded characteristics such as general characteristics, symptomatology, preoperative laboratory analyses, or radiological measurements are presented in Table 4.

We found a statistically significant difference between the male and female groups, in our series female patients present higher rate of complication than male (*p* = 0.003).

There were no statistically significant difference regarding age groups (*p* = 0.512), BMI (body mass index) (*p* = 0.074) or the symptomatology of the patients.

Analyzing the preoperative laboratory data, we found a lower hematocrit, hemoglobin, and albumin levels in the low-grade complication and high-grade complication groups compared to the group with no complication. Significantly higher CRP (C-reactive protein) was associated with a higher grade of complications by Clavien-Dindo classification (*p* = 0.043).

Among radiological measurements, the low-grade and high-grade complication groups showed a significantly lower right psoas muscle area, left psoas muscle area, total psoas muscle area, and psoas muscle index (*p* < 0.001 in all cases).

Spearmen’s correlation analysis was performed to examine association of psoas muscle index with preoperative laboratory markers. The PMI was significantly correlated with hematocrit (*r* = 0.30, *p* = 0.015), hemoglobin (*r* = 0.41, *p* = 0.003) and albumin (*r* = 0.33, *p* = 0.018). The correlation between CRP and PMI was negative and not significant (*r* = −0.22, *p* = 0.114).

Each variable that showed significant differences from the univariate analysis was used for multivariate logistic regression analysis. The dependent variables were represented by low grade complication (1–2 grade of Clavien Dindo Classification) and high grade complication (3–5 grade of Clavien Dindo Classification), the independent variables were the significant variables from the univariate analyses (female gender, hematocrit, hemoglobin, CRP, albumine, Na^+^, radiological parameters as right psoas muscle area, left psoas muscle area, total psoas muscle area, and psoas muscle index.

Logistic regression analysis of risk factors for occurrence of low-grade complication vs. no complications is represented by Table 5. This showed that the presence of a low-grade complication is independently associated with a lower- hematocrit (*p* = 0.013), hemoglobin (*p* = 0.002) and albumin level (*p* = 0.006). Among the radiological measurements a lower- right psoas area (*p* = 0.005), left psoas area (*p* = 0.006), total psoas area (*p* = 0.005) and psoas muscle index (*p* = 0.002) were independently associated with the appearance of low-grade complications. The female population also has a higher risk of developing a low grade complication than males (*p* = 0.001) (Table 5).

The presence of a high-grade complication was also independently associated with a lower- hemoglobin (*p* = 0.041), albumin (*p* = 0.038) and Na^+^ level (*p* = 0.022) in the logistic regression analysis of risk factors for occurrence of high-grade complication vs. no complications. Among the radiological measurements in this group a lower-right psoas area (*p* = 0.022), left psoas area (*p* = 0.014), total psoas area (*p* = 0.016) and psoas muscle index (*p* = 0.027) were independently associated with the appearance of high-grade complications (Table 5).

Summarizing the multivariate analyses of the preoperative laboratory markers and the radiological measurements, our results show that a lower-hemoglobin or albumin level as well as a smaller-right psoas area, left psoas area, total psoas area or psoas muscle index increases the probability of the appearance of a low- or high-grade complication.

## 4. Discussion

In recent years many studies highlighted the hypothesis that some well-defined perioperative nutritional and inflammatory factors can influence the clinical outcome of colorectal cancer patients [15,16,17]. Sarcopenia is closely related to generalized inflammation and induces skeletal muscle mass decrease and loss of function, associated with poor outcome, including mortality, length of hospital stays, falls, disability, risks of infection, postoperative complications, and poorer quality of life [18,19].

Recent systematic review and meta-analyses conducted by Giulia Bano et al. [19] have shown that an increased level of perioperative CRP is associated with sarcopenia, and there also is a direct association with the severity of complications [20]. These results are similar to our observation; in our study the elevated CRP level was associated with a higher grade of complication in Clavien-Dindo score.

The other powerful prognostic factor is the level of albuminemia as a marker of nutritional status. Some of the authors suggest the correction of the albumin level before surgery [21], this also proved to be significant in the occurrence of complications [22].

Regarding the preoperative laboratory values as hemoglobin, hematocrit, and albumin we have to summarize the potential complications in case of a modification of these, as it was demonstrated by Young Wan Kim et al. among patients over 80 years [23]. In our study a lower level of these laboratory results proved to be important to complication appearance.

However the male gender is considered as a risk factor for complication in colorectal cancer surgery [24], in our study the female gender seems to be associated with higher grade of complications, probably due to a higher incidence of comorbidities and to a small number of cases included in this study. Future studies with larger databases are needed to investigate potential differences between patients gender and the development of postoperative complications.

Following colorectal cancer surgery in elective conditions we had a similar complication rate to a study enrolled in 2015 by Jones KI et al. with the highest frequency in Grade 1–2 (low grade) by Clavien-Dindo score [11].

Radiological image analysis is being increasingly accessed to diagnose sarcopenia in patients with malignant and chronic disease due to the availability of CT and it is routinely performed perioperatively [25]. Sarcopenia assessed by psoas muscle index is a simple and practical method that should be helpful to estimate patient frailty and to predict surgical outcome not only for oncological patients. It’s utility was demonstrated in liver transplantation [26], complication prediction in resected non-small-cell-lung-carcinoma [27], a prognostic value in primary operable colorectal cancer [28] and advanced or recurrent colorectal cancer treated with regorafenib [29], ICU (Instesive Care Unit) stay predictor in trauma patients [30], gynecological malignant diseases [31] as in liver cirrhosis, when PMI is a predictive tool for long term mortality [32]. In a study conducted by Y. Zager et al. the radiological measurement of psoas area proved to be important as in Crohn’s disease among patients with bowel resection. This study also highlights the importance of psoas muscle area measurement in the recognition of postoperative complications [33].

It seems to be stronger relevance between malignancies and PMI from the point of view of the postoperative complications. However, our study included a reduced number of patients; these results are similar to important meta-analyses published by Guangwei Sun et al. This systematic review shows that patients with CRC and sarcopenia (defined at level L3 with skeletal muscle index (SMI) [34], like PMI) have prolonged hospital stay, increased morbidity and mortality in the postoperative period and these patients are more susceptible for infections [35].

Proved to be important also to define the outcome in hepatobiliary malignancies [36], another study by Hou JC et al. reflects the significant correlation between PMI and the early postoperative survival rate and incidence of complications among patients after liver transplantation. These facts are also similar to our results, when the lower PMI is associated with a higher grade of complication [37].

The importance of CT scan psoas density measurement is to predict the prevalence of the anastomotic leak and morbidity in colorectal cancer patients [38]. This study published by S.E. Tevis did not find psoas density as a prognostic factor, but the PMI has been demonstrated to be important in postoperative complication assessment. Complications are associated with poor postoperative outcomes and have been shown to affect long-term quality of life [39].

Serum albumin is a widely applicable preoperative risk assessment marker that has shown to predict poor outcomes in gastrointestinal, cardiac, thoracic and orthopedic surgery. In our study, serum albumin had a significant correlation with PMI, indicating that PMI has an important role in protein malnutrition state represented by hypoalbuminemia. This result reflects also to similar findings of a study conducted by Wilson D. Lo et al., where the psoas density was the predictor of outcome after enterocutaneous fistula repair [40]. However in our study the PMI and CRP values were negatively correlated but were statistically non-significant, any decrease of PMI could be considered to be associated with CRP elevation, the important marker of inflammation [41].

The skeletal muscle mass quantity defined by the PMI is a reliable prognostic factor in colorectal cancer surgery, but for more accurate assessment the muscle quality should also be measured. A recent study by Ojima et al. [42] demonstrates firstly that postoperative survival is closely associated also with the quality of skeletal muscle which is expressed by intramuscular adipose tissue content (IMAC). This method seems to be effective, because some patients present decreased muscle function and strength even though their muscular mass seems to be normal (by PMI) and probably this is correlated with declined muscle quality which is influenced by adipose tissue infiltration.

Studying these easily accesible variables as PMI, IMAC or visceral-to-subcutaneous adipose tissue area ratio (VSR), we can conclude that application of these indexes could be a keypoint in the perioperative management, but seems to be not effective in all malignant diseases. Based on several studies with patients undergoing liver resection for colorectal metastases the sarcopenia expressed by preoperative quality and quantity of skeletal muscle and visceral adiposity were not predictors of poor prognosis [43,44] and also sarcopenia is not a significant risk factor of overall and recurrence free survival [45].

Quantifying the material composition and quality of any anatomically defined skeletal muscle by CT scan is a novel method in patient specific therapy from a study group from Reykjavik University [46]. The aim of this biomedical image analysis is to improve surgical planning and to enhance clinical outcome. During this image analysis and tissue densitometry we can obtain a three dimensional muscle reconstruction to monitor and characterize sarcopenic and sequelae muscle degeneration [47,48]. Analyzing radio-densitometry distribution proved that implementing this tool could provide clinicians with information which can be useful in daily practice. The importance has been demonstrated in patient specific treatment as total hip arthroplasty, and also in construction of predictive tools for assessing the impact of lifestyle factors to predict the most common diseases as diabetes mellitus or hypertension [49,50].

We conducted this study to add some new information in order to understand the postoperative management of complications and to find the important factors which plays a key role in the occurrence of postoperative complications. The originality of this study consists in that it points out that we have to summarize some well-defined patient related laboratory and radiological parameters in order to obtain more accurate results.

The main limitation of the present study was the relatively small number of patients examined due to its prospective state. Our data came from a single institution in a couple of months tight before the COVID19 pandemic. This may not reflect the patient population or outcomes at other centers and it should be examined in the future assessing more details. Nonetheless, these are preliminary results and we would like to extend for other malignancies, to be more accurate in the patient assessment in the perioperative phase.

## 5. Conclusions

In our single center prospective study the PMI influence on the postoperative outcome is an important factor in colorectal surgery and appears to be a good overall predictor. A lower PMI is directly associated with a low or high grade of complication by Clavien-Dindo classification. Perioperative inflammatory and nutritional status evidenced by serum CRP and albumin level influences the presence of postoperative complications. Clinicians can use this method to identify patients who might benefit from additional interventions to reduce the complication occurrence and to improve prognosis.

## Figures and Tables

**Table 1:** Clavien Dindo postoperative complication scale (Dindo et al. 2004)**.**

*Grades*	*Definition*
*Grade I:*	Any deviation from the normal postoperative course without the need for pharmacological treatment or surgical, endoscopic and radiological interventions. Acceptable therapeutic regimens are: drugs as antiemetics, antipyretics, analgetics, diuretics and electrolytes and physiotherapy. This grade also includes wound infections opened at the bedside
*Grade II*	Requiring pharmacological treatment with drugs other than such allowed for grade I complications. Blood transfusions and total parenteral nutrition are also included.
*Grade III*	Requiring surgical, endoscopic or radiological intervention
*Grade III-a*	intervention not under general anesthesia
*Grade III-b*	intervention under general anesthesia
*Grade IV*	Life-threatening complication (including Central nervous system (CNS) complications)* requiring IC/ICU-management
*Grade IV-a*	single organ dysfunction (including dialysis)
*Grade IV-b*	multi organ dysfunction
*Grade V*	Death of a patient
*Suffix “d”*	If the patient suffers from a complication at the time of discharge, the suffix “d” (for ‘disability’) is added to the respective grade of complication. This label indicates the need for a follow-up to fully evaluate the complication

* Brain hemorrhage, ischemic stroke, subarrachnoidal bleeding, but excluding transient ischemic attacks (TIA); IC: Intermediate care; ICU: Intensive care unit.

**Table 2:** The frequency of cancer localization.

Characteristics	N (Number of Cases)	%
Cancer localization		
Rectal	29	56.9
Right colon	13	25.5
Sigmoid	6	11.8
Left colon	3	5.9

**Table 3:** Frequency of complications by Clavien-Dindo grade.

Postoperative Complications	N	%
Clavien-Dindo Complication Grade		
No complication (0)	30	58.8
Grade I	12	23.5
Grade II	4	7.8
Grade III	2	3.9
Grade IV	1	2.0
Grade V	2	3.9

**Table 4:** Patient characteristics by Clavien-Dindo complication grade.

Characteristic	All (*n* = 51)	Clavien-Dindo Complication Grade			
		0 ^a^ (*n* = 30)	1–2 ^b^ (*n* = 16)	3–5 ^c^ (*n* = 5)	*p*
**General characteristic**					
Age (years)	65 (56–71)	62 (55–71)	64 (59–69)	70 (69–71)	0.512
Sex, male/female, n (%)	31 (61)/20 (39)	24 (80)/6(20)	5(31)/11(69)	2(40)/3(60)	0.003
BMI (kg/m^2^)	26.3 (23.9–30.1)	27.6 (24.9–30.4)	23.7 (22.9–28.2)	25.6 (25.5–26.9)	0.074
**Symptomatology**					
Symptom duration (days)	3 (2–6)	3 (2–5)	3.5 (1–6)	3 (2–3)	0.517
Hemorrhage, n (%)	23 (45)	12 (40)	8 (50)	3 (60)	0.637
Loss in weight (kg)	5 (0–10)	3 (0–35)	6 (0–20)	8 (0–30)	0.941
Meteorism, n (%)	18 (25)	10 (33)	5 (31)	3 (60)	0.490
Diarrhea, n (%)	21 (41)	15 (50)	3 (19)	3 (60)	0.069
Constipation, n (%)	16 (31)	8 (27)	4 (25)	4 (80)	0.059
Abdominal pain, n (%)	23 (45)	13 (43)	7 (44)	3 (60)	0.780
Fatiguability, n (%)	18 (35)	8 (27)	8 (50)	2 (40)	0.284
**Preoperative laboratory**					
White blood cells (/μL)	7560 (6000–8560)	7000 (5500–8230)	7845 (6925–8525)	8030 (6200–8720)	0.454
Hematocrit (%)	39 (32–42)	42 (39–45)	33 (30–38)	32 (29–40)	0.002
Hemoglobin (g/dL)	12.6 (9.9–14.0)	13.5 (12.4–14.4)	9.9 (9.5–11.5)	10.8 (9.1–12.5)	0.001
Platelet (x10^3^/μL)	246 (208–299)	237 (193–267)	255 (228–299)	255 (208–423)	0.192
ALT (U/L)	14 (10–25)	14 (10–25)	15.5 (9–29.5)	13 (12–17)	0.915
AST (U/L)	17 (13–29)	15 (13–24)	17.5 (12–32.5)	18 (17–31)	0.563
Total protein (g/L)	69 (62–73)	69 (67–73)	67 (58–72)	55 (54–76)	0.318
Albumin (g/L)	40 (37–43)	41 (39–43)	37 (35–39)	33 (32–42)	0.008
Na^+^ (mEq/L)	140 (138–142)	141 (139–143)	140 (138–142)	136 (136–138)	0.019
K^+^ (mEq/L)	4.3 (4.0–4.6)	4.4 (4–4.7)	4.2 (4.1–4.5)	4.3 (3.9–4.4)	0.618
Creatinine (mg/dL)	0.77 (0.72–0.89)	0.78 (0.74–0.86)	0.73 (0.66–0.84)	1.07 (0.74–1.31)	0.105
ESR (mm/h)	37 (16–62)	30 (8–50)	46 (28–76)	24 (14–50)	0.121
CRP (mg/L)	0.7 (0.3–1.7)	0.4 (0.2–0.9)	1.0 (0.6–2.0)	2.7 (1.3–2.9)	0.043
**Radiological measurement**					
Right psoas area (cm^2^)	10.2 (8.5–11.9)	11.3 (10.2–12.7)	7.9 (6.7–8.9)	8.3 (7.8–11.1)	<0.001
Left psoas area (cm^2^)	10.3 (8.3–12.6)	12.3 (11.4–13.2)	8.3 (6.5–9.4)	8.5 (7.9–10.1)	<0.001
Total psoas area (cm^2^)	20.9 (16.7–24.3)	23.3 (21.1–25.8)	15.9 (13.6–18.3)	16.9 (15.6–21.2)	<0.001
Psoas muscle index (cm^2^/m^2^)	7.2 (5.9–8.2)	8.1 (7.3–8.6)	5.8 (5.1–6.1)	6.8 (5.9–7.3)	<0.001
Mean density (HU)	36.0 (30.5–40.3)	36.5 (32.0–40.5)	34.0 (39.8–41.0)	32.0 (28.0–37.5)	0.343

^a^ = No complication, ^b^ = Low grade (complication requiring minor intervention), ^c^ = High grade (complication requiring significant intervention). BMI = body mass index, ALT = alanine transaminase, AST = aspartate transaminase, ESR = erythrocyte sedimentation rate, CRP = C-reactive protein, HU = Hounsfield unit. Data are presented as median (interquartile range) or as frequencies and percentages.

**Table 5:** Logistic regression analysis of risk factors for occurrence of complication vs. no complications.

Low-Grade		
Predictors	OR(Odds Ratio) (95% CI)	*p* Value
**General characteristics**		
Female gender	8.80 (2.20–35.14)	0.001
**Preoperative laboratory markers**		
Hematocrit	0.87 (0.79–0.97)	0.013
Hemoglobin	0.55 (0.38–0.79)	0.002
CRP	1.35 (0.88–2.06)	0.159
Albumin	0.75 (0.61–0.92)	0.006
Na^+^	0.91 (0.72–1.15)	0.459
**Radiological measurements**		
Right psoas area	0.11 (0.02–0.50)	0.005
Left psoas area	0.18 (0.05–0.61)	0.006
Total psoas area	0.33 (0.16–0.72)	0.005
Psoas muscle index	0.05 (0.01–0.31)	0.002
**High–Grade**		
**Predictors**	**OR (95% CI)**	***p*** **Value**
**General characteristics**		
Female gender	6.00 (0.81–44.35)	0.076
**Preoperative laboratory markers**		
Hematocrit	0.90 (0.81–1.01)	0.080
Hemoglobin	0.64 (0.42–0.98)	0.041
CRP	1.56 (0.88–2.78)	0.125
Albumin	0.78 (0.61–0.98)	0.038
Na^+^	0.64 (0.44–0.93)	0.022
**Radiological measurements**		
Right psoas area	0.41 (0.19–0.88)	0.022
Left psoas area	0.45 (0.24–0.85)	0.014
Total psoas area	0.64 (0.44–0.91)	0.016
Psoas muscle index	0.26 (0.08–0.85)	0.027

## Data Availability

Data supporting the reported results can be found in the Archive of Surgical Clinic Mureș County Clinical Hospital, “G.E. Palade” University of Medicine, Pharmacy, Science and Technology of Târgu Mureş, Târgu Mureș, Romania.
